# Expression analysis of long non-coding RNAs in a renal ischemia-reperfusion injury model^1^

**DOI:** 10.1590/s0102-865020190040000003

**Published:** 2019-04-29

**Authors:** Qiu Tao, Wang Tianyu, Zhou Jiangqiao, Chen Zhongbao, Ma Xiaoxiong, Zhang Long, Zou Jilin

**Affiliations:** I PhD, Department of Organ Transplantation, Renmin Hospital, Wuhan University, Hubei, China. Conception and design of the study, acquisition and analysis of data, manuscript writing.; II PhD, Department of Organ Transplantation, Renmin Hospital, Wuhan University, Hubei, China. Design and supervised all phases of the study.; III Physician, Department of Organ Transplantation, Renmin Hospital, Wuhan University, Hubei, China. Technical procedures, acquisition of data.; IV Physician, Department of Organ Transplantation, Renmin Hospital, Wuhan University, Hubei, China. Manuscript preparation.

**Keywords:** Kidney Transplantation, Ischemia, Reperfusion, RNA, Messenger, Mice

## Abstract

**Purpose:**

To investigate the long non-coding RNAs (lncRNAs) profile on renal ischemia reperfusion in a mouse model.

**Methods:**

Microarray analysis was used to study the expression of misregulated lncRNA in a mouse model of renal ischemia reperfusion(I/R) with long ischemia time. Quantitative real-time PCR (qPCR) was used to verify the expression of selected lncRNAs and mRNAs.The potential functions of the lncRNA was analyzed by bioinformatics tools and databases.

**Results:**

Kidney function was impaired in I/R group compared to the normal group. Analysis showed that a total of 2267 lncRNAs and 2341 messenger RNAs (mRNAs) were significantly expressed in I/R group (≥2.0-fold, p < 0.05).The qPCR result showed that lncRNAs and mRNAs expression were consistent with the microarray analysis. The co-expression network profile analysis based on five validated lncRNAs and 203 interacted mRNAs showed it existed a total of 208 nodes and 333 connections. The GO and KEEG pathway analysis results showed that multiple lncRNAs are involved the mechanism of I/R.

**Conclusion:**

Multiple lncRNAs are involved in the mechanism of I/R.These analysis results will help us to further understand the mechanism of I/R and promote the new methods targeted at lncRNA to improve I/R injury.

## Introduction

 Occurrence of renal ischemia-reperfusion (I/R) injury affects the outcome of kidney transplantation, which has a direct relationship with the survival of the recipient. The delayed graft function (DGF) rate of kidney transplantation from donation after cardiac death (DCD) reached up to 40-70% and was significantly higher than that from brain death donors and living donors^1^. The main factor responsible for this difference was the prolonged warm ischemic time^2^. The I/R injury mechanisms in DCD kidney transplantation are multifactorial and may include oxidative stress, mitochondrial Ca2^+^ overload, inflammation, cell apoptosis, necrosis, loss of cell polarity, dedifferentiation and proliferation of viable cells, and disruption of the generation of free radicals. Long non-coding RNAs (lncRNAs) are typically longer than 200 nucleotides. It has been demonstrated that lncRNAs exert comprehensive effects on biological processes, such as transcription, translation, splicing, and intracellular and extracellular trafficking, and are associated with numerous diseases^3^. lncRNAs can interact with proteins, DNAs, and RNAs and regulate gene expression at various levels, including epigenetic, transcriptional, and post-transcriptional regulation^4^. However, the possible role of lncRNAs in I/R injury has not received much attention.

 Therefore, in this study, we used microarray analysis to analyze the lncRNA and mRNA expression differences in I/R model.The selected lncRNA and mRNA were verified by q-PCR. Gene Ontology(GO) analysis and Kyoto Encyclopedia of Genes and Genomes (KEGG) analysis were performed to predict possible biological processes and potential signal pathways. In addition, co-expression network of lncRNA-mRNA was clarified by coding/non-coding gene co-expression (CNC) analysis. 

## Methods

### Animal preparation and experimental design

 The research project was approved by the research ethics committee (NSCF 81400753). 

 Male C57BL/6 mice (body weight 250-300g) were obtained from the Experimental Animal Center of the Medical College of Wuhan University (Wuhan, China). The animals were maintained at the Central Animal Facility of Affiliated Renmin Hospital of Wuhan University according to standard guidelines, and experiments were conducted according to the guidelines of the Chinese Council for Animal Care. The mice were kept in an air-filtered, homoiothermal (20-22°C), and light-controlled room (light from 7 a.m. to 7 p.m.) and allowed free access to a standard diet.

### Sample collection

 Mice were separated into two groups.Group 1: control group (n = 5), in which mice were subjected to a right nephrectomy but without the induction of a left renal ischemia, and Group 2: I/R group (n = 5), in which mice were subjected to a right nephrectomy and left renal ischemia for 45 min followed by a reperfusion period of 24 h. All mice were anesthetized with intraperitoneal pentobarbital (50 mg/kg) and were killed via decapitation. Mice were placed on an electric heating pad to maintain their body temperature at 37°C. Their kidney tissues were removed and frozen in liquid nitrogen followed by storage at −80°C prior to analysis.

### Kidney function test and hematoxylin and eosin staining

 After the 24-h reperfusion period, blood samples were collected from the inferior vena cava and centrifuged to determine the concentration of creatinine (Cr). The Cr was measured in the blood using standard techniques with an Olympus AU 2700 Analyzer (Olympus Optical Co., Ltd., Tokyo, Japan).

 After a 24 h reperfusion, the left kidney was excised. Then, the kidney tissue was fixed with 10% phosphate-buffered formalin, paraffin embedded, and sectioned to a thickness of 4 mm according to a standard procedure. Sections were deparaffinized and gradually hydrated before they were examined by hematoxylin and eosin (HE) staining. Morphological assessments were performed by an experienced renal pathologist who had not been informed of the experimental protocol.

### Microarray analysis

 Gene microarray analysis was performed on 5 pairs of kidney tissues from I/R group and normal group to detect differentially expressed lncRNA and mRNA. Approximately 35,923 lncRNAs and 24,881 coding transcripts were detected by the Arraystar Mouse LncRNA Microarray V3. The tissue preparations and microarray hybridization were performed using the Agilent Gene Expression Hybridization Kit (Agilent Technology Inc., USA). Then, the arrays were scanned using the Agilent Microarray Scanner and were finally analyzed using the Agilent Feature Extraction software. Differentially expressed transcripts were identified by fold-change screening at a threshold of ≥2-fold and a p-value of <0.05^5^. Pathway analysis was used to study the significant signaling pathways of the differentially expressed genes. GO analysis was used to explore the biological roles of the aberrantly expressed mRNAs, which included to three domains-molecular function, biological process, and cellular component. 

### qRT-PCR validation

 The kidney tissue collected for lncRNA microarray analysis were used for quantitative real-time polymerase chain reaction (qRT-PCR) validation.According to the instructions of the product, SuperScript III reverse transcriptase(Invitrogen, Grand Island, NY, USA) is used to reverse transcribe the total RNA into cDNA. The qRT-PCR was performed with the Applied Biosystems ViiA 7 RT PCR System and 2× PCR Master Mix. The PCR conditions consisted of: incubation at 95°C for 10 min, followed by 40 cycles of 95°C for 10 s and 60°C for 1 min. The relative expression levels of lncRNAs were calculated using the 2^−ΔΔCt^ method and were normalized against β-actin. The primers for each gene are listed in Table 1. The data represent the means of three experiments.

**Table 1:** Primers designed for qRT-PCR validation of candidate lncRNAs and mRNAs.

Name lncRNA/mRNA	Primer	Tm(ºC)	Product length(bp)
GAPDH(MOUSE)	F:5’ CACTGAGCAAGAGAGGCCCTAT3’ R:5’ GCAGCGAACTTTATTGATGGTATT3’	60	144
ENSMUST00000124572	F:5’ GGTTAAAGCAACGGACAGAG 3’ R:5’ TGGCAAAGTCCTTACCACAG 3’	60	96
ENSMUST00000180989	F:5’AACCCGACAATGTAAGGACC3’ R:5’ TGTAAAACCGCACAAGGCT3’	60	200
ENSMUST00000147219	F:5’CCAGTTGAGGAGACAGGGAA3’ R:5’ TGGCTAGAGCAGGGGATTA3’	60	92
ENSMUST00000097928	F:5’TTGGAATGCCTTGGAGATG3’ R:5’ GTTGGTTGTCACCGTTGCT 3’	60	145
uc007mos.1	F:5’GCTGAGGCTTTGATGTTGC3’ R:5’ TCCAGTTCTTGACGCTCTTCT 3’	60	115
ENSMUST00000145410	F:5’CTACCTGGACCCCAATACAAC3’ R:5’ ACCCAAGGCAAGTCACAAA3’	60	181
NR_040589	F:5’GGACAGGATTTGGATTTCGA3’ R:5’ TGACAGACCACCATAACAGACA3’	60	184
ENSMUST00000139773	F:5’CAGTGCTCAAGAGACTCAGAAAA3’ R:5’ AACAGGTGCTGGTCAAAGG3’	60	259
ENSMUST00000169128	F:5’ CCATCTAATGCCCTTTTCTG 3’ R:5’ GCTTGTTCTGTATGTACTGGACC 3’	60	150
NM_025684	F:5’ CCTCCGAGACCTGAAACATC 3’ R:5’ CCCTCCAGTGCCTTGAAAT 3’	60	279
AK078749	F:5’ CGCTAATTCTTCCTCCGTG 3’ R:5’ TGATTGGTCCGCACTTCTT 3’	60	135
NM_028746	F:5’ ACTCGGTGTCATTTCCCTCA 3’ R:5’ TTCTTCAGCTCCCCTGCTAT 3’	60	189
NM_013913	F:5’ AACAGAGGCGAACATACAAGTG3’ R:5’ CGTTGAAGTCCTGTGAGCCAT 3’	60	139
NM_020013	F:5’ GGAGGATGGAACAGTGGTAGGC3’ R:5’ AGGCTTTGACACCCAGGATTTG3’	60	104
NM_009114	F:5’ ATACTGGGCTTACACTGCTCTT3’ R:5’ CTGTGCTTCCACCATTTGTC3’	60	193

### GO annotations and KEGG analysis

 We conducted a GO analysis to construct a meaningful annotation of genes and gene products. The ontology covers the domains of biological processes, cellular components, and molecular functions. The −log10 (p-value) denotes enrichment score representing the significance of GO term enrichment among differentially expressed genes. Pathway analysis was performed to explore the significant pathways in differentially regulated gene profiles according to KEGG. Also, the −log10 (p-value) denotes an enrichment score showing the significance of the pathway correlations.

### Construction of a co-expression network with GO and KEGG analysis

 In order to identify the interaction between differentially expressed lncRNA and mRNA, we constructed the co-expression network with the verified lncRNA and related mRNA on the basis of correlation analysis. Cytoscape software was applied into construct the network between lncRNA and mRNA, while the pearson’s correlation coefficients should not less that 0.992. In the figure of CNC, mRNAs were represented by a red node, while lncRNAs were represented by a green node.

### Statistical analyses

 Statistical analyses were performed using Graphpad Prism 5.0 (San Diego, CA, USA). Results were expressed as mean ± standard error of the mean. The t-test was used to analyze the differences between the normal control and I/R group data in this study. Spearman’s correlation analysis was used to detect the relationship between lncRNAs and mRNAs.A p-value of <0.05 and a fractional disappearance rate of <0.05 were used as thresholds to define markedly enriched GO terms/pathways.

## Results

### Kidney function test and HE stain

**Figure 1:**
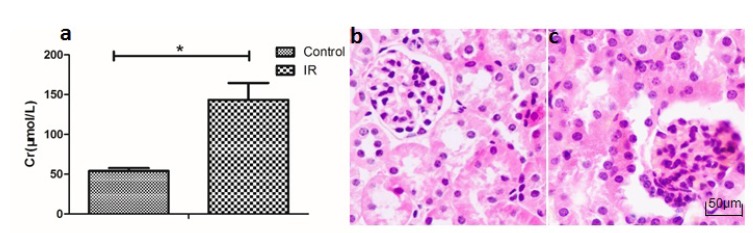
Renal function was impaired in the I/R group when compared with the normal control group. **a.** The level of serum Cr is higher in the I/R group; “*” represents p<0.05. **b, c.** HE staining in kidney tissue of the I/R (**b**) and control groups (**c**), showing I/R features in the tubular cells of the I/R group, including tubular necrosis, renal tubular expansion, and renal tubular epithelial cellular microvilli disappearance (magnification ×200).

 Compared with the normal control group, we observed increased levels of serum creatine in I/R group. HE staining of the kidney tissues showed that renal tubular cells in I/R group presented I/R injury features, including tubular necrosis, renal tubular expansion, and renal tubular epithelial cellular microvilli disappearance (Fig. 1).

### lncRNA and mRNA expression analysis in renal I/R injury

 To explore the difference in the expression of lncRNA and mRNA in I/R model, microarray analysis was used to assess the expression levels. We identified 2267 significantly dysregulated lncRNAs in the I/R mice, while 1180 were upregulated, while 1087 were downregulated (≥2.0-fold, p < 0.05). Table 2 presents the 50 most significantly differentially expressed lncRNAs. 

**Table 2:** Top 25 up and down expressed lncRNA in microarray analysis.

Seq ID	p value	Fold change	Regulation	Strand	Relationship	Chr	GeneSymbol
NR_003548	2.35187E-08	109.4887219	up	+	intergenic	3	Sprr2g
uc007mos.1	1.4733E-09	47.7396161	up	-	exon sense-overlapping	11	Cyth1
humanlincRNA1590+	3.31079E-08	44.0254584	up	+	intergenic	5	humanlincRNA1590
ENSMUST00000097928	1.94377E-06	41.8941913	up	+	natural antisense	7	Gm10574
ENSMUST00000147219	1.02731E-08	39.3596471	up	-	exon sense-overlapping	2	Lcn2
ENSMUST00000171867	1.41682E-07	39.0995403	up	-	intergenic	14	Gm9890
ENSMUST00000138796	1.00309E-08	35.7404452	up	-	exon sense-overlapping	16	BC100530
NR_073362	8.0896E-09	32.0782555	up	-	intergenic	14	1700091H14Rik
ENSMUST00000167016	1.296E-10	30.2506392	up	-	intergenic	14	Gm17030
AK053244	1.19946E-05	30.1399734	up	-	intergenic	13	AK053244
ENSMUST00000163699	1.53946E-08	29.9253704	up	-	intergenic	14	Gm3719
AK078011	1.5941E-07	29.2616428	up	-	intergenic	14	AK078011
uc009oqw.1	6.091E-10	27.1826111	up	+	intronic antisense	9	AK077358
ENSMUST00000146254	3.28551E-07	25.6293449	up	-	exon sense-overlapping	11	Cd300lf
ENSMUST00000149944	1.63907E-04	25.0363506	up	+	exon sense-overlapping	17	Fpr2
TCONS_00022944	1.63913E-08	23.5285727	up	-	intergenic	4	TCONS_00022944
ENSMUST00000122073	4.7E-12	23.0889698	up	-	intergenic	4	Gm12551
ENSMUST00000149186	7E-13	22.5034757	up	+	intergenic	5	BC028471
AK078752	2.10663E-06	22.1980481	up	+	intergenic	3	AK078752
ENSMUST00000175727	2.52131E-08	22.1525562	up	+	exon sense-overlapping	2	AA467197
ENSMUST00000169043	1.1936E-07	20.8414822	up	-	intergenic	14	Gm17159
NR_028066	2.00597E-07	20.2041383	up	-	intergenic	11	Gm4926
NR_045891	1.61E-11	20.0657937	up	-	intergenic	13	1700016G22Rik
ENSMUST00000163480	8.719E-10	19.6386359	up	-	intergenic	14	Gm3123
ENSMUST00000146415	2.36414E-08	19.2413828	up	-	exon sense-overlapping	4	Ubxn10
AK015307	6.02535E-08	126.3849319	down	+	bidirectional	1	AK015307
ENSMUST00000120915	1.29281E-07	61.9993283	down	-	intergenic	3	Gm12400
uc009kit.1	2.20769E-08	54.6821234	down	+	intergenic	7	BC024386
ENSMUST00000121475	4.523E-10	44.2610944	down	-	intergenic	3	Gm12399
ENSMUST00000132986	1.306E-10	41.1148477	down	-	intergenic	11	Gm12326
ENSMUST00000120693	8.7904E-09	31.1848562	down	-	intergenic	3	Gm12431
ENSMUST00000137279	6.623E-10	29.4712609	down	+	exon sense-overlapping	13	Akr1c21
uc007szw.1	1.89775E-08	28.0074467	down	+	intergenic	14	AK136780
ENSMUST00000152146	2.03281E-08	23.9584478	down	-	exon sense-overlapping	4	C8a
ENSMUST00000145410	2.27236E-08	22.5110902	down	+	natural antisense	9	Gm16010
AK041352	6.3143E-09	19.4262943	down	-	intergenic	13	AK041352
TCONS_00000485	2.93747E-07	18.6743919	down	-	intergenic	1	XLOC_001255
mouselincRNA1395+	1.51813E-08	17.9271355	down	+	intergenic	7	mouselincRNA1395
ENSMUST00000154598	3.01441E-08	17.1697404	down	-	exon sense-overlapping	2	Gatm
ENSMUST00000131133	1.43599E-07	16.513081	down	-	intergenic	4	Gm12354
ENSMUST00000155540	1.25951E-08	16.2209832	down	-	intergenic	3	5730437C11Rik
uc007ana.1	1.23943E-07	16.0098561	down	+	intergenic	1	AK050085
ENSMUST00000133801	3.42096E-07	14.7898681	down	+	exon sense-overlapping	7	Aspdh
AK045907	4.7641E-09	14.3347262	down	-	intronic antisense	5	AK045907
humanlincRNA2217-	2.94943E-07	14.1063714	down	-	intergenic	4	humanlincRNA2217
NR_040589	6.761E-10	13.8372003	down	+	natural antisense	3	6330410L21Rik
NR_102276	9.9E-12	13.2454412	down	+	intergenic	7	AI314278
AA189272	5.1804E-05	13.2368177	down	+	intergenic	2	humanlincRNA1141
uc008yin.1	3.89772E-08	12.9396912	down	+	intergenic	5	AK020506
ENSMUST00000144755	8.3739E-08	12.8921193	down	+	exon sense-overlapping	15	Ugt3a2

SeqID: lncRNA name. P value:P value calculated from unpaired t-test. Fold Change: the absolute ratio (no log scale) of normalized intensities between two groups (IR vs Control). Chr: chromosome no. which lncRNA is transcribed. Strand: the strand of chromosome which lncRNA is transcribed; ‘+’ is sense strand of chromosome, ‘−’ is antisense strand of chromosome. Relationship: sense exon overlap^: the LncRNA’s exon is overlapping a coding transcript exon on the same genomic strand; sense intron overlap^: the LncRNA is overlapping the intron of a coding transcript on the same genomic strand; antisense_exon_overlap^: the LncRNA is transcribed from the antisense strand and overlapping with a coding transcript; antisense_intron_overlap^: the LncRNA is transcribed from the antisense strand without sharing overlapping exons;”bidirection^: the LncRNA is oriented head to head to a coding transcript within 1000 bp; intergenic^: there are no coding transcripts within 30 kb of the LncRNA; others^: means other LncRNAs. GeneSymbol: lncRNA gene symbol.

**Figure 2:**
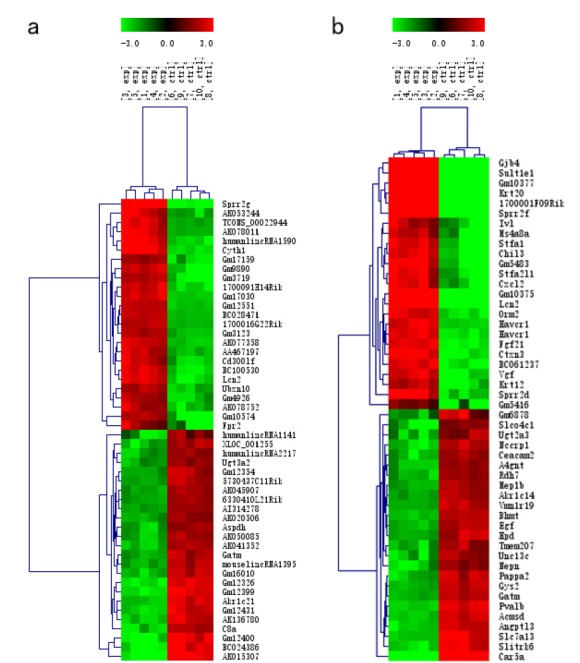
Heat map and hierarchical clustering of the 50 most significantly differentially expressed lncRNAs (**a**) and mRNAs (**b**) between the I/R and control groups. The data are depicted as a data matrix, in which each row represents one lncRNA (mRNA) and each column represents one sample. The relative lncRNA (mRNA) expression follows the color scale at the top. Red represents high relative expression, and green represents low relative expression; −3.0, 0, and 3.0 are fold changes in the corresponding spectrum. The magnitude of deviation from the median is represented by the color saturation.

**Figure 3:**
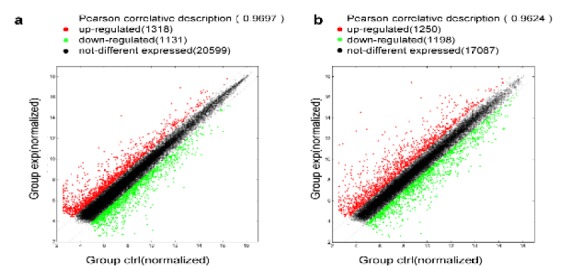
Scatter plot of lncRNA (**a**) and mRNA (**b**) expression variation between the I/R and control kidney samples. The values shown on the X-axis and Y-axis are normalized signal values for each sample (log2 scale). The dark lines are fold-change lines (the default fold-change value given is 2.0). The green dot and red plots showed an expression fold-change of >2.0 between the two samples compared.

 Heat map and hierarchical clustering of the 50 most significantly differentially expressed lncRNAs expression patterns in different samples (Fig. 2a). All the variation in lncRNA expression between the I/R and normal control groups is shown using a scatter plot (Fig. 3a). At the same time, 2341 significantly dysregulated mRNAs were identified in the I/R group, while1166 were upregulated, while 1175 were downregulated (≥2.0-fold, p<0.05). Table 3 presents the 50 most significantly differentially expressed mRNAs. Heat map and hierarchical clustering of the 50 most significantly differentially expressed mRNA expression patterns in different samples (Fig. 2b). All the variation in mRNA expression between the I/R and control groups is shown using a scatter plot (Fig. 3b).

**Table 3:** Top 25 up and down expressed mRNAs in microarray analysis.

GeneSymbol	P-value	Fold Change	Regulation	seqname	strand	chrom
Sprr2f	1.9366E-09	1148.181643	up	NM_011472	+	chr3
1700001F09Rik	2.64E-11	442.6745614	up	NM_027940	-	chr14
Sult1e1	1.26948E-06	427.0273786	up	NM_023135	-	chr5
Krt20	1.1E-12	425.3504883	up	NM_023256	-	chr11
Gm10377	7.795E-10	384.4778351	up	NM_001244671	-	chr14
Gjb4	3.63597E-06	255.815718	up	NM_008127	-	chr4
Gm10375	1.275E-10	135.8332007	up	NM_001098269	-	chr14
Lcn2	1.9E-12	87.6107745	up	NM_008491	-	chr2
Sprr2d	1.62662E-05	81.1614053	up	NM_011470	+	chr3
Vgf	5.89051E-06	74.1857763	up	NM_001039385	+	chr5
Gm5483	1.96007E-06	70.9697136	up	NM_001082547	+	chr16
BC061237	5.57972E-08	62.5468169	up	NM_198677	+	chr14
Ctxn3	8.5312E-09	60.5381104	up	NM_001134697	+	chr18
Chil3	2.73372E-07	54.7387104	up	NM_009892	-	chr3
Stfa1	1.88196E-06	51.3837601	up	NM_001082543	+	chr16
Fgf21	1.228E-10	49.9305516	up	NM_020013	-	chr7
Havcr1	3.4119E-09	45.0066413	up	NM_001166631	+	chr11
Orm2	2.40498E-08	41.6527274	up	NM_011016	+	chr4
Ms4a8a	6.13093E-05	41.6072802	up	NM_022430	-	chr19
Krt12	7.77949E-07	37.0094785	up	NM_010661	-	chr11
Stfa2l1	1.56089E-06	36.7724705	up	NM_173869	+	chr16
Havcr1	1.5051E-09	35.743044	up	NM_134248	+	chr11
Cxcl2	4.13451E-06	35.369706	up	NM_009140	+	chr5
Gm5416	1.8353E-04	34.6385309	up	NM_001082542	+	chr16
Ivl	2.12671E-04	34.0747508	up	NM_008412	-	chr3
Car5a	1.913E-10	97.8555546	down	NM_007608	-	chr8
Slitrk6	3.88686E-07	42.1200797	down	NM_175499	-	chr14
Pvalb	6.27E-11	34.7214021	down	NM_013645	-	chr15
Slc7a13	1.36165E-08	33.6931965	down	NM_028746	+	chr4
Angptl3	1.90038E-08	29.4659423	down	NM_013913	+	chr4
Acmsd	3.843E-10	28.087504	down	NM_001033041	+	chr1
Nccrp1	1.31903E-07	24.4742272	down	NM_001081115	-	chr7
Pappa2	2.19209E-07	23.125535	down	NM_001085376	-	chr1
Bhmt	2.9E-12	21.4082897	down	NM_016668	-	chr13
Gys2	1.5504E-09	21.171212	down	NM_145572	-	chr6
Gatm	2.206E-10	19.2441547	down	NM_025961	-	chr2
Egf	1.5411E-09	18.9547528	down	NM_010113	-	chr3
Vmn1r19	3.176E-09	18.8127661	down	NM_134182	+	chr6
Unc13c	4.3197E-06	18.5175514	down	NM_001081153	-	chr9
Hpd	4.49995E-08	18.3616339	down	NM_008277	-	chr5
Nepn	1.81913E-06	17.6920316	down	NM_025684	+	chr10
Slco4c1	3.77297E-06	17.2959218	down	NM_172658	-	chr1
Ceacam2	9.98525E-07	17.2607985	down	NM_001113369	-	chr7
Mep1b	6.6751E-09	15.9869509	down	NM_008586	+	chr18
A4gnt	2.49574E-08	15.9408784	down	NM_001077424	+	chr9
Tmem207	1.41886E-06	15.5512338	down	NM_001101640	-	chr16
Akr1c14	9.6481E-09	15.0846971	down	NM_134072	+	chr13
Rdh7	5.5158E-08	14.8091606	down	NM_001150749	-	chr10
Ugt2a3	9.93539E-06	14.5629187	down	NM_028094	-	chr5
Gm6878	1.9959E-04	14.4289555	down	NM_001037931	-	chr14

GeneSymbol: mRNA gene symbol.P value:P value calculated from unpaired t-test. Fold Change: the absolute ratio (no log scale) of normalized intensities between two groups(IR vs Control). SeqID: mRNA name.Chr: chromosome no. which lncRNA is transcribed. Strand: the strand of chromosome which lncRNA is transcribed; ‘+’ is sense strand of chromosome, ‘−’ is antisense strand of chromosome.

### Validation of the microarray data using qRT-PCR

**Figure 4:**
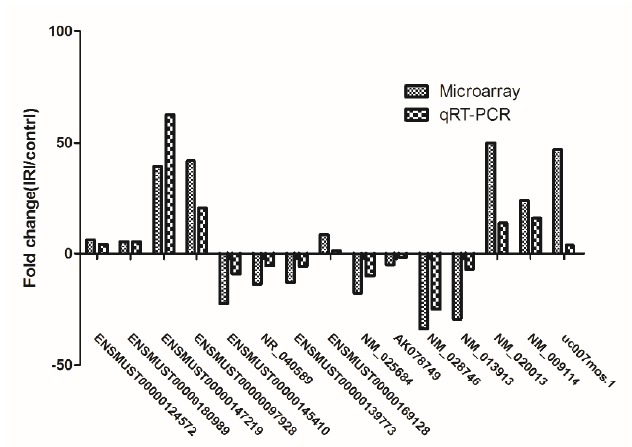
The differential expression of lncRNAs and mRNAs was validated by quantitative real-time PCR (qRT-PCR). The data show that expression levels of lncRNAs ENSMUST00000145410, NR_040589, ENSMUST00000139773, NM_025684, and AK078749, along with mRNAs NM_028746 and NM_013913 were downregulated, while expression levels of lncRNAs ENSMUST00000124572, ENSMUST00000180989, ENSMUST00000147219, ENSMUST00000097928, ENSMUST00000169128, uc007 mos.1, and mRNAs NM_020013 and NM_009114 were upregulated in kidney tissue samples from I/R mouses when compared with the control mouses. The heights of the columns in the chart represent fold changes. The qRT-PCR results were consistent with the microarray data.

 To validate the result of mRNA and lncRNA from microarray analysis, we performed the qRT-PCR assay.Ten mRNA and lncRNA were randomly selected to perform the qRT-PCR.The results showed that the expressions of lncRNA ENSMUST00000124572, ENSMUST00000180989, ENSMUST00000147219, ENSMUST00000097928, uc007mos1 and ENSMUST00000169128 were upregulated, whereas those of ENSMUST00000145410, NR_040589, ENSMUST00000139773, and AK078749 were downregulated (Fig. 4). Meanwhile, when compared with the control group, three target mRNAs (NM_028746, NM_013913, and NM_025684)were found to be downregulated in the I/R group, whereas NM_020013 and NM_009114 were upregulated (Fig. 4). These results are consistent with those of the microarray analysis (Fig. 4), thereby confirming the validity of the microarray results. The finding provides compelling evidence that these lncRNAs and mRNAs could be implicated in the pathogenesis of renal I/R injury.

### GO analysis and KEGG pathway analysis

**Figure 5:**
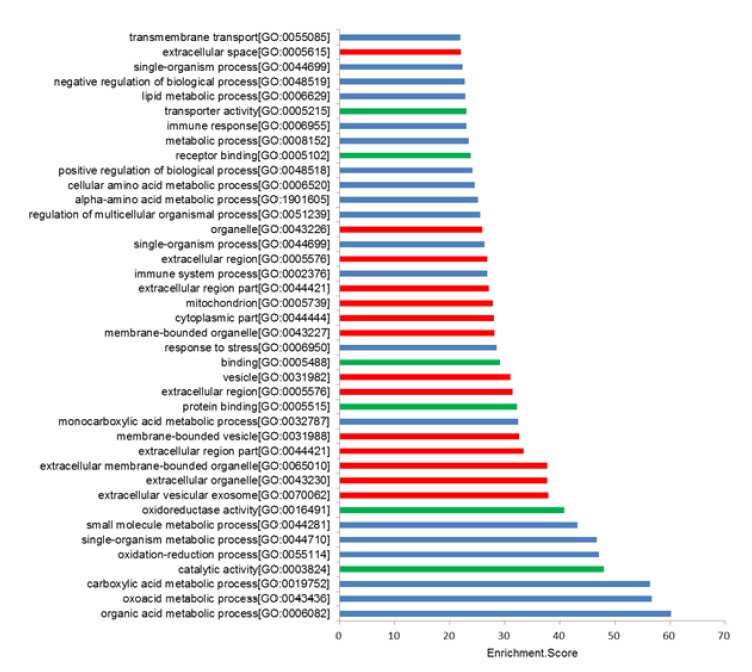
The 40 most significant GO terms for differences in co-expressed lncRNA genes in I/R animals and controls. GO enrichment analysis provided a controlled vocabulary to describe co-expressed genes of differentially expressed lncRNAs. The ontology covered three domains: biological process (*blue*), cellular component (*red*), and molecular function (*green)*.

 The GO defines concepts/classes used to describe gene function, and relationships including three aspects molecular function, biological process and cellular component. In this study the GO term analysis indicates that the most enriched GO terms targeted by mRNAs co-expressed with lncRNAs were organic acid metabolic process (ontology: biological process, GO:006082), catalytic activity (ontology: molecular function, GO:0003824), and extracellular vesicular exosome (ontology: cellular component, GO:0070062) (Fig. 5).

**Figure 6:**
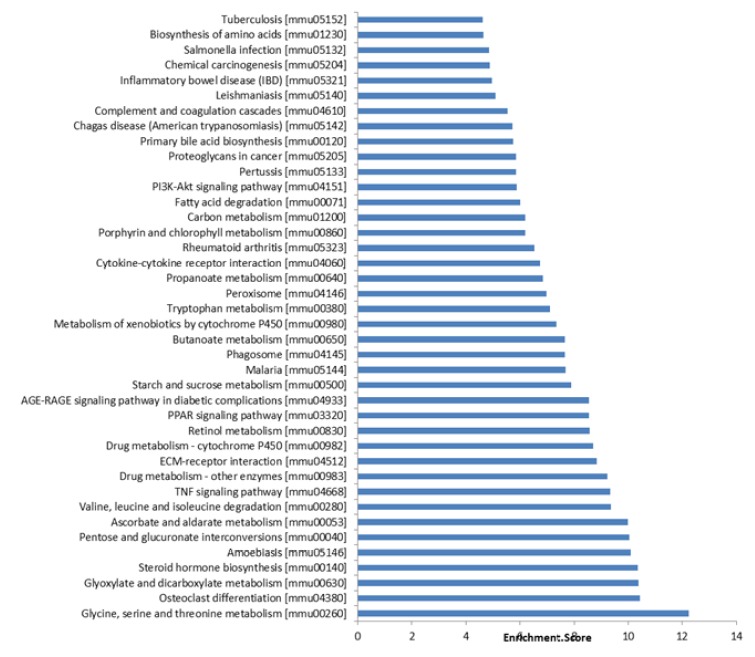
The 40 most significant pathways for differences in lncRNA genes co-expressed in I/R injury animals and controls.

 Furthermore, we applied the KEGG tool to explore pathway maps on the molecular interaction, reaction and relation networks .the results indicated that mRNAs co-expressed with lncRNAs were involved in the regulation of glycine, serine, and threonine metabolism; TNF signaling pathway; AGE-RAGE signaling pathway in diabetic complications; cytokine-cytokine receptor interaction; ECM-receptor interaction; PI3K-Akt signaling pathway; and others. The 40 most significant KEGG pathways are listed in Figure 6.

### lncRNA-mRNA CNC network analysis

**Figure 7:**
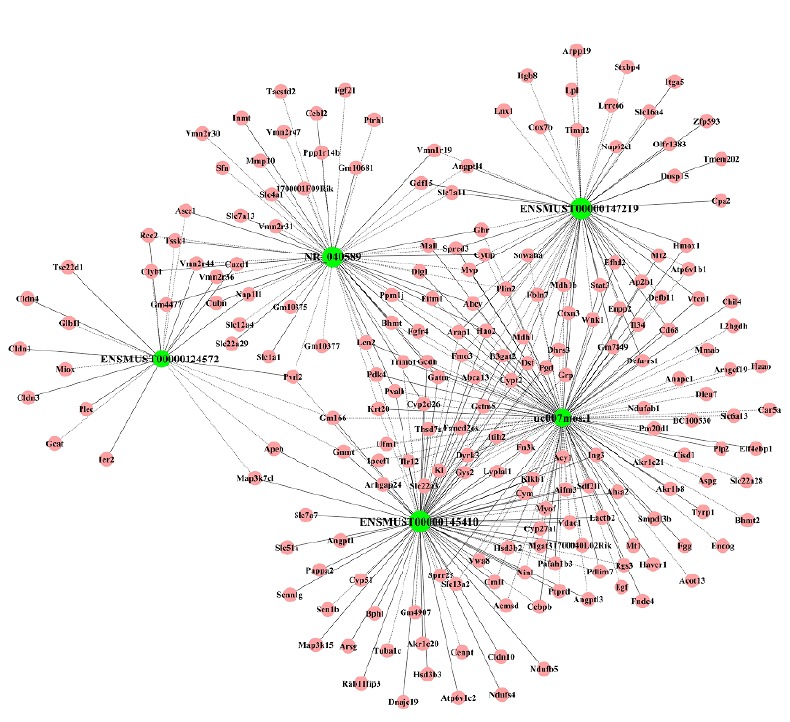
lncRNA-mRNA network analysis. Green nodes represent dysregulated lncRNAs, whereas red nodes represent dysregulated mRNAs. The dotted lines between lncRNAs and mRNAs indicate a negative correlation, whereas solid lines indicate a positive correlation.

 LncRNA is involved in the occurrence and development of I/R, but lncRNA mainly play functions by regulating the expression of mRNA, so we further analyzed the CNC network of lncRNA and mRNA. The 5 differentially expressed lncRNAs that were validated by qRT-PCR (uc007 mos.1, ENSMUST00000147219, ENSMUST00000124572, ENSMUST00000145410, and NR_040589) with 203 interacting mRNAs were used to construct the co-expression network. This co-expression network was consisted of 208 nodes and 333 connections, of which 89 were negative and 244 were positive interactions. The resulting network revealed that uc007 mos.1 is correlated with 119 mRNAs, ENSMUST00000147219 is correlated with 54 mRNAs, ENSMUST00000124572 is correlated with 22 mRNAs, ENSMUST00000145410 is correlated with 81 mRNAs, and NR_040589 is correlated with 57 mRNAs (Fig. 7) .

**Figure 8:**
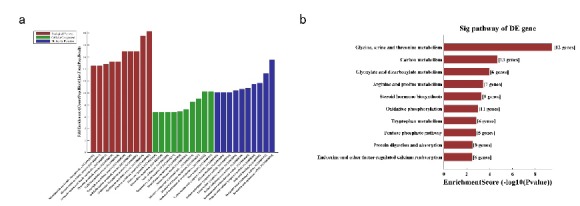
GO analysis and pathway analysis with which targeted genes are correlated. **a.** According to the enrichment score levels, the results showed that mRNAs were enriched for organic acid metabolic process (ontology: biological process, GO: 0004930), oxidoreductase activity (ontology: molecular function, GO:0016491), and extracellular exosome (ontology: cellular component, GO:0070062). **b.** The result of KEGG pathway analysis with the targeted gene. Most of genes were predicted to interplay with glycine, serine, and threonine metabolism.

 GO and KEGG analyses based on the results of the co-expression network were performed. According to the enrichment score level, the result showed that the targeted mRNA was focused on the organic acid metabolic process (ontology: biological process, GO:0004930), oxidoreductase activity (ontology: molecular function, GO:0016491), and extracellular exosome (ontology: cellular component, GO:0070062) (Fig. 8a).

 In addition, most genes predicted by KEGG analysis were involved with glycine, serine, and threonine metabolism (pathway ID: mmu00260) (Fig. 8b). These results show that randomly selected lncRNA samples are very representative.

## Discussion

 lncRNAs encompass a large and diverse class of transcribed RNA molecules that are longer than 200 nucleotides and do not encode proteins^6^. In recent years, more and more studies have shown that 1ncRNA participate in the occurrence and development of many diseases through a variety of mechanisms. lncRNAs contain complementary binding sites to micro RNAs (miRNAs) and serve as endogenous miRNA sponges, forming an lncRNA-miRNA axis to regulate cell processes, such as apoptosis, necrosis, and autophagy. lncRNAs can be categorized as sense, antisense, bidirectional, intronic, or intergenic, depending on their position with regard to protein coding genes^7^ ^,^ ^8^. LncRNAs interact with components of the cellular machinery, including protein, DNA, RNA and chromatin remodelling complexes, to regulate the expression of target genes.

 Recent discoveries suggest that lncRNAs participate in renal processes and play pathogenic roles in kidney diseases^9^ ^-^ ^11^. Animal studies have linked lncRNAs to diabetic nephropathy, glomerular disease, acute renal allograft rejection, renal cell carcinoma, acute kidney injury, and hypertension. The regulation of lncRNAs expression will become novel targets for the treatment of kidney diseases^4^ ^,^ ^6^ ^,^ ^12^.

 Although the role of non-coding RNA in renal ischemia reperfusion has been reported^13^, we tried to use a longer time model of thermal ischemia reperfusion (ischemia time 45 min), which may lead to cell necrosis and inflammation and get the different resuls. We explored whole transcriptome profiles in a mouse model of I/R using microarray analysis and bioinformatics analysis. The results of serum creatine levels and HE pathological staining verified the reliability of the I/R model (Fig. 1). By microarray analysis, it has been identified 2267 significantly dysregulated lncRNAs in the I/R group, as well as 2341 significantly deregulated mRNAs in the I/R mouse (Figs. 2 and 3). These data provide the groundwork for a comprehensive analysis of potential lncRNAs involved in I/R.

 Many studies have demonstrated that lncRNAs play an important role in AKI and other kidney diseases^3^ ^,^ ^7^ ^,^ ^14^. In the mouse model of renal I/R injury model, significant RANTES expression was observed in the renal tubular cells of wild type mice. RANTES-deficient mice showed improved renal function with reduced acute tubular necrosis, serum Cr levels, infiltration of inflammatory cells, and cytokine expression compared with the wild type mice^15^. The four specific target genes-ankyrin repeat and SOCS box 3, cation transport regulator homolog 2, peroxisomal membrane protein 11B, and trans-acting transcription factor 5 had been identified as being similarly associated with differentially expressed lncRNAs to regulate blood pressure and kidney disease^16^.In addition, we identified several of the dysregulated lncRNAs and mRNAs, based on qRT-PCR validation, and the results confirmed the microarray analysis findings to some extent (Fig. 4).

 Recent studies have implicated lncRNAs can become potential biomarkers for related diseases in remodeling and dysfunction after I/R^17^. Thus, circulating or urinary lncRNAs may be fascinating novel biomarkers, which noninvasively reflect intra nuclear processes. And the lncRNA (e.g.AK139328 and lncRNA-PRINS) in the pathogenesis of I/R may therefore be markers of intracellular processes than currently established conventional biomarkers^11^ ^,^ ^18^. lncRNAs are strongly altered in the urine of patients with acute rejection, and urinary RP11-354P17.15-001 may serve as a novel biomarker of acute kidney rejection and predict loss of kidney function^19^. Plasma levels of circulating lncRNAs (TapSAKI, also known as MGAT3-AS1), could predict survival in patients with dialysis-dependent AKI. Arid2-IR is a novel lncRNA that functions to promote NF-κB-dependent renal inﬂammation^20^ ^,^ ^21^. lncRNA-H19 expression was significantly upregulated in TGF-β2-induced HK-2 cell fibrosis and unilateral ureteral obstruction-induced renal fibrosis in vivo^22^.

 Furthermore, to explore the potential functions of the differentially expressed lncRNAs identified in this study, GO and KEGG pathway analysis were performed using coding genes associated with significantly differentially expressed lncRNAs. GO analysis revealed that these lncRNAs are involved in biological processes such as organic acid metabolic process, catalytic activity, and extracellular vesicular exosome. KEGG pathway analysis indicated the enrichment of multiple pathways including glycine, serine, and threonine metabolism; cytokine-cytokine receptor interaction and PI3K/Akt signaling pathway (Figs. 5 and 6) . These results are consistent with previous studies of renal I/R.lncRNAs could serve as sponges to bind to miRNAs to regulate gene expression. The interactive networks of lncRNAs that regulate mRNAs reveal the important role of lncRNA function, which has biological significance^23^ ^,^ ^24^. The CNC network was performed to analyze the indicated lncRNA in this study, the result also showed these lncRNAs intersect multiple mRNAs and play multiple function in different pathways (Figs. 7 and 8). lncRNAs play multiple functions in different types of kidney cells, including renal tubular cells, endothelial cell, and podocytes. Long *et al.* ^6^ reported that the lncRNA Tug1 contributes to CKD development. lncRNAs also exert the biological functions in many cellular components, such as the extracellular exosome, extracellular vesicle, and extracellular organelle^7^ ^,^ ^25^. All these suggest the great potential of lncRNA in the occurrence and development of renal diseases.

## Conclusions

 lncRNA-mRNA expression was detected in the mouse model of kidney I/R by high-throughput microarray analysis. The expression profile showed that lncRNAs participate in several biological processes in renal I/R injury. Further research is needed to explore the potential role of lncRNAs in renal I/R injury.
